# TMEM120A and B: Nuclear Envelope Transmembrane Proteins Important for Adipocyte Differentiation

**DOI:** 10.1371/journal.pone.0127712

**Published:** 2015-05-29

**Authors:** Dzmitry G. Batrakou, Jose I. de las Heras, Rafal Czapiewski, Rabah Mouras, Eric C. Schirmer

**Affiliations:** 1 Wellcome Trust Center for Cell Biology, University of Edinburgh, Edinburgh, Scotland, United Kingdom; 2 Institute for Materials and Processes, University of Edinburgh, Edinburgh, Scotland, United Kingdom; Brunel University, UNITED KINGDOM

## Abstract

Recent work indicates that the nuclear envelope is a major signaling node for the cell that can influence tissue differentiation processes. Here we present two nuclear envelope trans-membrane proteins TMEM120A and TMEM120B that are paralogs encoded by the *Tmem120A* and *Tmem120B* genes. The TMEM120 proteins are expressed preferentially in fat and both are induced during 3T3-L1 adipocyte differentiation. Knockdown of one or the other protein altered expression of several genes required for adipocyte differentiation, *Gata3*, *Fasn*, *Glut4*, while knockdown of both together additionally affected *Pparg* and *Adipoq*. The double knockdown also increased the strength of effects, reducing for example *Glut4* levels by 95% compared to control 3T3-L1 cells upon pharmacologically induced differentiation. Accordingly, TMEM120A and B knockdown individually and together impacted on adipocyte differentiation/metabolism as measured by lipid accumulation through binding of Oil Red O and coherent anti-Stokes Raman scattering microscopy (CARS). The nuclear envelope is linked to several lipodystrophies through mutations in lamin A; however, lamin A is widely expressed. Thus it is possible that the TMEM120A and B fat-specific nuclear envelope transmembrane proteins may play a contributory role in the tissue-specific pathology of this disorder or in the wider problem of obesity.

## Introduction

In the last 30 years obesity and an associated increase in diabetes has become a worldwide problem with over 1.5 billion adults being classified as overweight (body mass index ≥ 25) in 2008 by the World Heath Organization [1]. Obesity is associated with increased levels of white adipose tissue (WAT) and can reflect either an increase in adipocyte cell number or in the amount of fat stored per cell, typically in the form of lipid droplets, as the genetics of obesity are extremely complex. Genome-wide association studies have identified roughly 75 genetic variants that increase the risk of obesity, though many of these are not drivers of adipogenesis *per se* [2,3]. The process of adipogenesis itself is extremely complex involving over 100 factors already identified with new factors being added with considerable frequency [4]. Among the primary transcriptional drivers are C/EBP, PPARγ, and KLF proteins while the enormity of signaling cascades include sonic hedgehog, TGFβ, FGF, Wnt and insulin pathways [4].

The wide range of functions recently found to occur at the nuclear envelope (NE), the double membrane system surrounding the nucleus, indicates that it is a major signaling node for the cell [5,6]. Separate from the transport function of the nuclear pore complexes, several NE transmembrane proteins (NETs) add an additional layer of regulation to a variety of well-known signaling pathways, including some of those known to be involved in adipogenesis. For example, knockout of the NET emerin results in changes in transcription profiles relating to 10 signaling pathways in heart [7,8], including the Wnt and TGFβ pathways also involved in adipogenesis [9,10] and MAPK and JNK kinase cascades. The NET MAN1 separately affects Smad/BMP/TGFβ signaling in bone morphogenesis, presumably through sequestration of Smads at the NE [11–13]. By additional recruitment of the phosphatase PPM1A, MAN1 is further able to inactivate the bound Smads [14].

Some more direct NE effects on adipogenesis have also been described. Though less striking than in heart, emerin influences on the Wnt signaling pathway also appear to affect adipogenesis [15]. Lamin A, an intermediate filament protein of the NE, has been linked to Dunnigan-type familial partial lipodystrophy, characterized by loss of subcutaneous fat from limbs and trunk with simultaneous fat accumulation in the face and neck and typically associated with insulin resistance and diabetes mellitus [16,17]. Lamin A mutations also cause mandibuloacral dysplasia type A [18] and Seip syndrome [19] that also exhibit defects in adipose tissue and diabetes. Although defects in fat storage are not observed in the lamin A-associated Atypical Werner premature aging syndrome, diabetes mellitus is included in its associated symptoms [20]. As lamin A is widely expressed, the adipogenic effects might be related to its ability to bind SREBF1 [21], an important factor in adipocyte differentiation that induces the master transcription factor PPARγ and also influences the induction of lipid biosynthesis in response to insulin [22,23]. Knockdown of lamin A in 3T3-L1 pre-adipocytes actually mildly enhances some characteristics of adipogenesis [24], suggesting that, as in the case of MAN1 with Smads in TGFβ signaling, the binding to lamin A sequesters SREBF1 away from its targets that promote adipogenesis.

Though lamin A and emerin both contribute to adipogenesis, both are widely expressed. A recent series of proteomic studies in different tissues has identified many tissue-specific or tissue-restricted NETs [24–28]. One of these, originally numerically named NET29 from a list of NE proteins identified by proteomics [27], is expressed preferentially in adipose tissue. Therefore we sought to determine if it, like lamin A and emerin, contributes to adipocyte differentiation and/or metabolism.

NET29 is encoded by the *Tmem120A* gene. Humans and mice also have a paralog of this gene encoded by *Tmem120B* and we will henceforth refer to the protein gene products by their gene names, TMEM120A and B. We previously confirmed NET29/TMEM120A as an inner nuclear membrane protein [29] and so first tested if this was also the case for the *Tmem120B* gene product. TMEM120B also targeted to the NE and it resisted detergent pre-extraction, verifying it as a *bona fide* NET. We proceeded to test both proteins for their contribution to adipogenesis using the well-established 3T3-L1 *in vitro* adipogenesis system [30–32]. Both the *Tmem120A* and *Tmem120B* genes are induced very early in 3T3-L1 adipogenesis. Reduction of TMEM120A mildly interfered with adipogenesis, as measured by expression of adipogenic markers and accumulation of lipid droplets. Reduction of TMEM120B had a more severe effect on adipogenesis, while reducing both TMEM120A and B exhibited an additive effect. Surprisingly, overexpression of TMEM120A in the double-knockdown background rescued adipogenesis more prominently than overexpression of TMEM120B. TMEM120A and B are the first NE proteins described that are preferentially expressed in fat and play significant roles in adipogenesis.

## Materials and Methods

### Plasmids

The *Tmem120A* and *Tmem120B* coding sequences were PCR amplified from a mouse 3T3-L1 cDNA library using the following primers: *Tmem120A*.f GCATATGACCAAACTCCAGGCCA, *Tmem120A*.r GCTCGAGCATCTGGTTTTCCAGCTCCTG, *Tmem120B*.f GCATATGACCTCCCTGCAGACGC, *Tmem120B*.r GCTCGAGAATGTTGGCGGTCATCTGTTG. The human *Tmem120A* variant was cloned from IMAGE clone IRAT 6201334. The human *Tmem120A* gene was used for the antibody testing,rescue and pull-down experiments: all other experiments were performed with the mouse genes. All were cloned into the pEGFP-N2 (Clontech) vector.

For knockdowns Sigma MISSION clones TRCN0000247877 targeting CDS of *Tmem120A*, TRCN0000253427 targeting 3’ UTR of *Tmem120B*, non-target shRNA SHC003 or empty vector SHC001 were used. Note that TRCN0000253427 (TMEM120B shRNA) also partially knocked down *Tmem120A* in differentiated cells. The sequence, however, in theory should not have an effect because there was no significant homology here between the two genes: TRCN0000253427 target sequence, CCTGTCCCAGCTCCCTATTTA; equivalent *Tmem120A* sequence, CCTGCTTCCGTTCCTCTTCTT.

For rescue experiments, human TMEM120A and mouse TMEM120B were cloned into pRRLSIN.cPPT.PGK-GFP.WPRE plasmid [33] cut with *BamHI* and *SalI* restriction enzymes (replacing GFP) using isothermal Gibson assembly. This lentiviral vector does not have a selection marker, but as lentiviruses were used to transduce cells the efficiencies should be high. Corresponding amplification primers were as follows: *Tmem120A*.Hs.f, CCGAATCACCGACCTCTCTCCCCAGGGGATGCAGCCCCCGCCC; *Tmem120A*.Hs.r, CACAAATTTTGTAATCCAGAGGTTGATTGTCAATCCTTCTTGCTCCCGTGC; *Tmem120B*.Mm.f, CCGAATCACCGACCTCTCTCCCCAGGGGATGTCCGGCCAGCTGG; *Tmem120B*.Mm.r, CACAAATTTTGTAATCCAGAGGTTGATTGTCATGGCTGCTTTGTCTTGTTTC.

For generating SBP constructs, SBP was amplified from the pTrAP vector [34] and subcloned via BamHI and NotI sites into the pEGFP-N2 vector, replacing GFP with SBP. To generate the ΔNTD mutant of human TMEM120A-SBP (lacking amino acids 2–105), site directed mutagenesis was done using the NEB Q5 mutagenesis kit strategy with minor modifications. Briefly, non-phosphorylated primers were used (forward 5’-GGATTGTACCTGAGCCTGGTTCTG and reverse 5’-CATGGGTCGAGATCTGAGTCCG) in a PCR reaction with Phusion polymerase and TMEM120A-SBP plasmid. The resulting PCR product was gel-purified and simultaneously phosphorylated and ligated using PNK and T4 DNA ligase (NEB), followed by transformation into chemically competent DH5α cells. All the constructs used in this study were sequenced at the regions of interest using Sanger DNA sequencing.

### Cell culture, transfections and stable cell line generation

3T3-L1 mouse pre-adipocyte cells (ATCC) were maintained in DMEM supplemented with 9% v/v FBS and streptomycin/ampicillin with special care taken to ensure that no subsections of plates were ever allowed to achieve confluency as the cells have been reported to exhibit reduced differentiation potential when confluency is reached outside of directed differentiation. Also to avoid reduced differentiation potential cells were never used past passage 15. To induce adipogenesis, the medium was replaced two days after reaching confluency with the adipocyte medium (pre-adipocyte medium supplemented with 10 μg/ml insulin, 1 μM dexamethasone and 0.5 mM 3-isobutyl-1-methylxanthine). This fully supplemented medium was replaced after two days and after four days post-induction, the medium was replaced again with the pre-adipocyte medium supplemented with just insulin (10 μg/ml final) and this medium was freshly replaced every two days thereafter. For transient transfections, jetPRIME reagent (Polyplus) was used following the protocol of the manufacturer. To generate stable knockdown cell lines, low passage 3T3-L1 cells were infected with VSV-G lentiviruses produced with Sigma MISSION shRNA plasmids based on the PLKO.1 vector and selected with 2 μg/ml puromycin (Sigma). In this manner the passage numbers were maintained low to prevent late passage deficiencies in differentiation. To generate rescue cell lines, the double-knockdown cell line was transduced with retroviruses produced with pRRLSIN.cPPT.PGK-GFP.WPRE based constructs.

### Protein sample preparation, antibodies and Western blots

Fresh mouse tissues were obtained by dissection and snap frozen in liquid nitrogen for storage at -80°C. The tissues were ground in liquid nitrogen and total protein was isolated using TRIzol following the manufacturer protocol. Protein samples were prepared from 3T3-L1 cells similarly after directly lysing in TRIzol. Protein concentration was quantified using the Pierce BCA Protein Assay Kit. For antibody specificity testing, transiently transfected HT1080 cells were lysed directly in protein sample buffer (50 mM Tris pH 8.0, 1% SDS, 1 mM EDTA, 10% glycerol v/v, trace amounts of Coomassie Brilliant Blue) and sonicated. Prior to running, all samples were supplemented with 50 mM DTT and incubated for 10 min at 65°C. Proteins resolved by SDS-PAGE were transferred onto nitrocellulose membranes (Odyssey 926–31092), blocked with 6% w/v non-fat milk in PBS supplemented with 0.1% v/v Tween-20 and stained with primary antibodies for 1 h at room temperature or overnight at 4°C. Primary antibodies used in this study were: anti-NET29/TMEM120A (Millipore 06–1018 to peptide TRQKKRLQELALALKKCKPS), anti-Histone H3 (Abcam 10799), anti-β-actin (Sigma A1978), anti-GFP polyclonal rabbit antibody generated to full-length protein produced in bacteria in the Schirmer lab and mouse monoclonal anti-SBP antibody (kind gift from Dr. Kumiko Samejima) [34]. Corresponding secondary HRP conjugated antibodies (rabbit—GE Healthcare NA934-1ML, mouse—Jackson ImmunoResearch 715-035-151) were then incubated for 45 min at room temperature and reacted with ECL (Amersham) shortly prior to exposure to Hyperfilm MP (Amersham). Alternatively, for quantitative Western blots, IRDye 800CW Donkey anti-Rabbit IgG and IRDye 680RD Donkey anti-Mouse IgG were incubated for 1 h at room temperature and detected using Odyssey LI-COR infrared imager.

### Quantitative reverse transcription PCR (qRT-PCR)

Total RNA was isolated from liquid nitrogen ground tissues and culture cells using TRIzol reagent following the manufacturer protocol and quantified using NanoDrop 2000. 5 μg of total RNA was reverse transcribed using 1 μM poly(dT)_21_ primer in a reaction with SuperScript II Reverse Transcriptase (Life Technologies) following the manufacturer protocol. qRT-PCR was carried out on a LightCycler480 using SYBR Green I Master Mix (Roche). Primer sequences were as follows: *Adipoq*.f TGTCTGTACGATTGTCAGTGG, *Adipoq*.r AGTAACGTCATCTTCGGCATG, *Fasn*.f CTCAAGATGAAGGTGGCAGAG, *Fasn*.r GGTCGGTGGCTGTGTATTC, *Gata3*.f GCCCCTTATCAAGCCCAAG, *Gata3*.*r* GTCCCCATTAGCGTTCCTC, *Glut4*.f GTAACTTCATTGTCGGCATGG, *Glut4*.r GTAACTTCATTGTCGGCATGG, *Pparg*.f TCACAAGAGCTGACCCAATG, *Pparg*.r ATGCTTTATCCCCACAGACTC, *Tmem120A*.f GAAAACCAGATGAAAGAGCGC, *Tmem120A*.r GTGAAGGAGATGACGATGAGG, *Tmem120A*.Hs.f GGAGAACCAGATGAAAGAGCG, *Tmem120A*.Hs.r GAAGGAGATGAGGATGAGGATG, *Tmem120B*.f TGCTCGTGTGGTATTACTGC, *Tmem120B*.r AGGAACGTGGAGACATAATGG, *Tpm1*.f GCTGGATCAGACTTTACTGGA, *Tpm1*.r TCTCGGCAGATGTTTCAGTG, *Srebf1*.f GAACCTGACCCTACGAAGTG, *Srebf1*.r TTTCATGCCCTCCATAGACAC, *Sun1*.f CCTAGAACTGGAACTGAACCT, *Sun1*.r TCATCCTTGCTCACGAACC.

Crossing points of the SYBR Green fluorescence in the qRT-PCR reactions were called using Second Derivative Maximum method of LightCycler480 software. To compare knockdown specificity in non-differentiated adipocytes, the Pfaffl method was used [35] with values from cells transduced with empty vector taken as the control sample. For tissue specificity and differentiation, expression was quantified using absolute quantification (Second Derivative Maximum method) and arbitrarily choosing standard samples. For tissue specificity, tissues expressed the lowest levels of *Tmem120* were chosen (skin for *Tmem120A* and testis for *TMM120B*), while for differentiation, samples from day 8 after induction were chosen as standards.

Statistical analysis was addressed by performing ANOVA followed by post-hoc Tukey HSD correction using statistical software R [36].

### Immunofluorescence microscopy

Cells were washed once with PBS and either directly fixed with 3.7% formaldehyde or first incubated in pre-extraction buffer (20 mM HEPES pH 7.4, 110 mM KOAc, 2 mM Mg(OAc)_2_, 1 mM EDTA, 2 mM DTT, 0.2% Triton X-100) for 1 min, followed by fixation with 3.7% formaldehyde for 10 min. After two washes with PBS, cells were stained with DNA stain Hoechst 33342 and membrane dye DiOC6 for 10 min, followed by brief wash with PBS, and mounted in Fluoromount G (Southern Biotech).

Images were obtained using a Nikon TE-2000 microscope equipped with a 1.45 NA 100x objective, Sedat quad filter set, PIFOC Z-axis focus drive (Physik Instruments), and CoolSnapHQ High Speed Monochrome CCD camera (Photometrics) run by Metamorph image acquisition software. Image stacks (0.2 μm steps) were deconvolved using AutoquantX. Micrographs were saved from source programs as. tif files and prepared for figures using GIMP.

### Lipid staining

Cells were washed twice with PBS and fixed with 3.7% formaldehyde (Sigma) in PBS for 10 min, followed by a brief wash with PBS. LipidTOX (Molecular Probes H34476) at 1:500 dilution in PBS was incubated on cells for 30 min along with 5 μg/ml Hoechst 33342 (Molecular Probes), followed by a brief wash in PBS.

Oil Red O working solution was prepared by dissolving 0.35% w/v Oil Red O (Sigma) overnight in isopropanol followed by passage through a 0.45 μm filter. Two volumes of this solution were mixed with one volume of water by gentle rocking overnight at 4°C. The solution was passed through a 0.2 μm filter. Cells were fixed with 4% formaldehyde in PBS for 60 min, washed once with PBS, once with water and once with 66% isopropanol. The working Oil Red O solution was added to the fixed cells for 1 h, washed three times with water and imaged on a standard tissue culture microscope fitted with a Nikon CoolPix 5000 digital camera. The plates were air-dried and the dye was extracted with isopropanol. Absorbance of the extracts was measured at 500 nm wavelength in a CECIL CE2041 Spectrophotometer. Statistical analysis was addressed by performing ANOVA followed by post-hoc Tukey HSD correction using the free statistical software R [36].

### CARS microscopy

Coherent Anti-Stokes Raman Scattering (CARS) [37] is a non-invasive, label free imaging technique based on Raman spectroscopy in which image contrast is inherently generated from the molecular vibrations of the chemical bonds. CARS microscopy uses two pulsed lasers operating at different frequencies, whose difference frequency is matched to the required vibrational frequency of a particular molecular bond. Due to the nonlinear nature of the process, the signal is greatly amplified by around five orders of magnitude over traditional Raman spectroscopy, offering new capabilities of imaging at high speeds with high sensitivity and three-dimensional (3-D) optical sectioning.

The experimental setup used in this study has been described elsewhere [38]. Briefly, a pump laser beam at frequency ω_p_ and a Stokes laser beam at frequency ω_s_ generated from a mode-locked Nd:YVO4 laser source (Pico-Train, High-Q Laser, Hohenems, Austria) are collinearly overlapped and tightly focused into a sample using an Olympus XLPlan N 25x 1.05 N.A. water immersion objective. A laser canning confocal inverted optical microscope (C1 Eclipse, Nikon BV, Amsterdam, Netherlands) is used to acquire images. Appropriate sets of custom short-pass and bandpass (BP650) filters (Chroma, Rockingham, USA) were used to suppress the radiation at the laser wavelengths and selectively transmit the CARS signal.

For CARS imaging, the pump and Stokes beams were tuned to ω_p_ = 12244 cm^-1^ (λ_p_ = 816.7 nm) and ω_s_ = 9398 cm^-1^ (λ_s_ = 1064 nm) to excite the Raman frequency at ω = 2845 cm^-1^ corresponding to the vibration of CH_2_ in lipids, giving a CARS signal at ω_as_ = 15085 cm^-1^ (λ_as_ 662.9 nm). Short-pass filters (945SP, 775SP, 700SP), and a band-pass filter (660/13, FWHM = 13 nm) were used to collect the CARS signal.

### Pulldowns

3T3-L1 cells were transfected with combinations of plasmids encoding mouse *Tmem120A* and *Tmem120B* with in-frame GFP at their C-termini and either full length human *TMEM120A* fused to SBP (C-terminal tag) or its truncated variant lacking the first 105 amino acids. The next day the cells (3 million/transfection) were washed once with PBS, once with TEN_250_ buffer (10 mM Tris HCl pH 7.4, 1 mM EDTA, 250 mM NaCl) and lysed with 0.5 ml of TEN_250_ buffer containing 1% (w/v) Triton X-100. The clarified extract was incubated rotating for 45 min at room temperature with streptavidin magnetic beads (Roche, 30 μl/transfection). The beads were washed three times for 1, 5 and 10 min with 1 ml of TEN_250_ buffer containing 1% Triton X-100, followed by a 5 min wash with 0.1 ml TEN_250_ buffer containing 1% Triton X-100 and 0.1% (w/v) SDS. The bound material was eluted with 50 μl of sample buffer supplemented with 50 mM DTT at 65°C and run on SDS-PAGE for Western blotting. GFP fusions were detected using anti-GFP antibody, truncated Tmem120A-SBP was detected using anti-SBP antibody, and full length TMEM120A-SBP fusion was detected using anti-TMEM120A antibody.

### Bioinformatics

Sequence alignments were performed using ClustalW2 and transmembrane domains were predicted with TMHMM v2.0. ER retrieval sequence was detected using PSort II.

## Results

### TMEM120B is a bona fide NET

**Fig 1:**
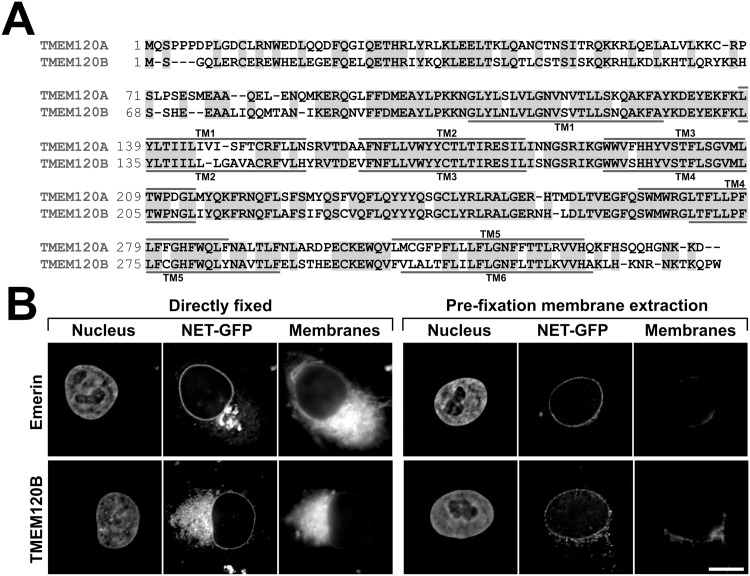
Paralog TMEM120B and its targeting to the NE. (A) Sequence alignment between mouse TMEM120A and TMEM120B. Identical residues are highlighted with gray shading and predicted transmembrane spans (TM) are indicated. (B) Targeting of TMEM120B to the NE. Left panels, directly fixed HT1080 cells expressing TMEM120B fused to GFP or positive control emerin-GFP (NET-GFP) stained for DNA (Nucleus) or with the membrane dye DiOC6 (Membranes). Right panels, equivalent cells were extracted with 0.2% Triton X-100 prior to fixation. This treatment should remove membranes and proteins not strongly bound to structures such as the nucleo/cytoskeleton and chromatin. Scale bar, 10 μm.

TMEM120A was originally identified in a proteomic study investigating the composition of rodent liver NEs, where it was designated NET29 in a numerical list of NETs [27]. A subsequent study investigating the targeting of these NETs further confirmed it in the inner nuclear membrane by super resolution microscopy and its resistance to a pre-fixation detergent extraction, characteristic of proteins associating with the nuclear lamina [29]. TMEM120A has a paralog, TMEM120B, the product of *TMEM120B* gene. The gene duplication happened relatively early in higher evolution—sharks and rays already have two copies of the gene while flies do not. The mouse TMEM120A and B proteins are about 70% identical (Fig 1A) and the human TMEM120A and B are similarly identical.

We have tested whether TMEM120B also targets to the NE. For this, TMEM120B was exogenously expressed as a fusion to GFP and transfected into HT1080 human fibrosarcoma cells. The fused protein displayed, typical to other overexpressed NETs, rim-like staining with some also accumulating in the ER (Fig 1B, left panels). Pre-fixation extraction with detergent normally removes transmembrane proteins unless they are bound to more stable structures. For many NETs including TMEM120A [29], the NE pool was resistant to pre-extraction with detergent, typically indicating interaction with the intermediate filament lamin polymer or chromatin. TMEM120B similarly resisted the pre-fixation extraction with detergent (Fig 1B, right panels). Membrane staining confirming membrane extraction is shown in the third panels on each side. It should be noted, however, that TMEM120B contains a predicted ER membrane retention signal. Although such predictions are not highly reliable (http://psort.hgc.jp/psort/helpwww2.html#er), nonetheless it is quite possible that a separate fraction of the endogenous protein exists in the ER.

### TMEM120A and B are expressed preferentially in fat tissue

**Fig 2:**
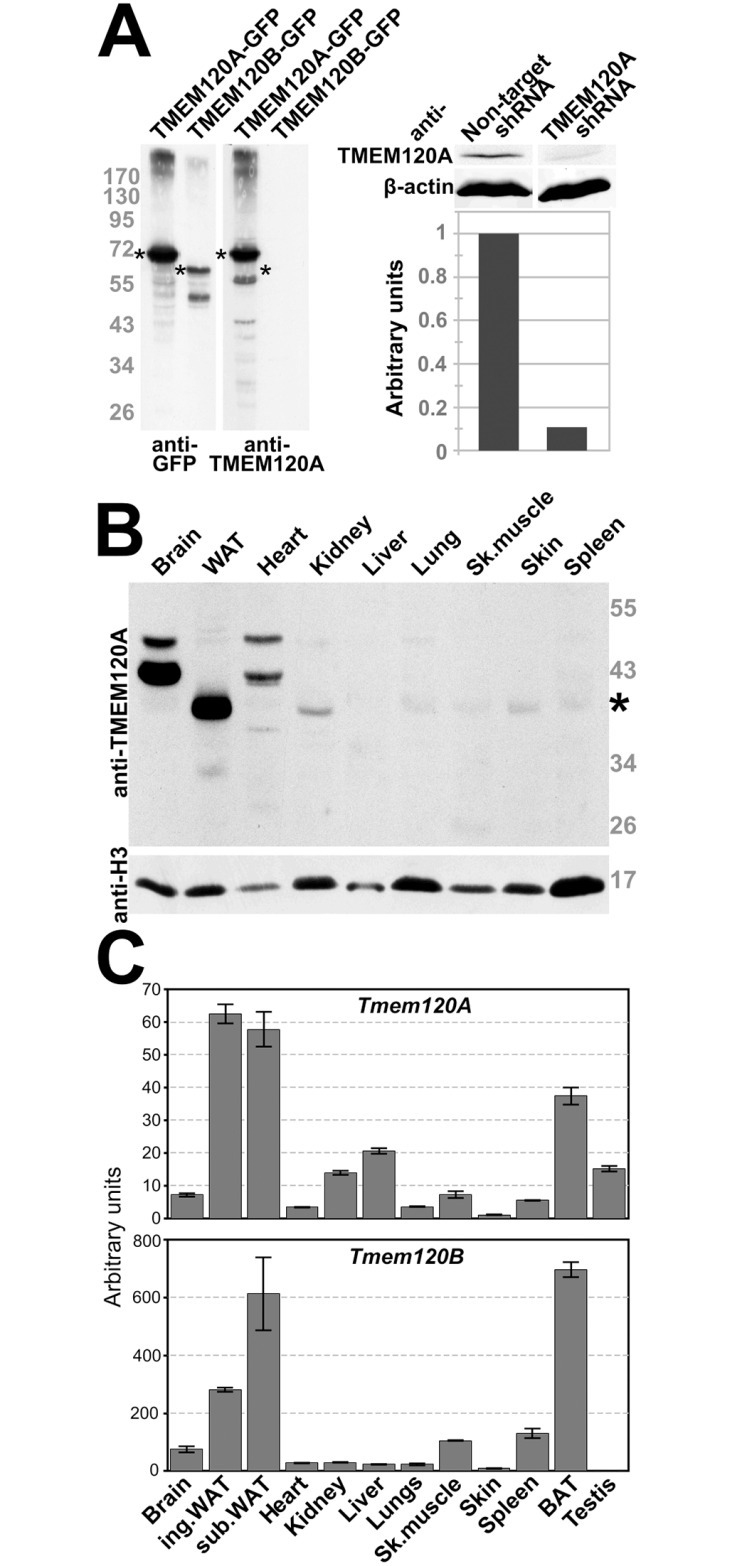
Preferential expression of TMEM120A and B in fat. (A) TMEM120A-specific antibodies. Antibodies generated in rabbit to amino acid residues 52–71 were tested against lysates from HT1080 cells expressing TMEM120A and B as GFP fusions and 6 days differentiated 3T3-L1 stably expressing either non-target shRNA or TMEM120A-specific shRNA. The left panel is HT1080 cells exogenously expressing TMEM120-GFP fusions reacted either with the GFP antibody or with the TMEM120A antibody; asterisks denote TMEM120A and B GFP fusion proteins. The right panel, top, is quantitative Western blot of 3T3-L1 cell lysates from the shRNA knockdown and controls reacted with either the anti-TMEM120A or anti-β-actin antibody; bottom, quantification of the TMEM120A signal from the above blot, normalized to β-actin. (B) Lysates were generated from freshly isolated mouse tissue and reacted on Western with the TMEM120A antibodies. Of the tissues tested, WAT expressed by far the strongest a band for the expected size of TMEM120A; however, two bands larger than the size for any predicted splice variant were also strongly expressed in brain and to a lesser degree in heart. Thus it is unclear if these are TMEM120A variants. (C) Total RNA was extracted from mouse tissues and subjected to qRT-PCR using specific primers for *Tmem120A* and *Tmem120B*. The data is expressed as the fold-increase over the lowest level (skin for Tmem120A and testis for Tmem120B). Error bars indicate the standard deviation for three technical repeats.

An antibody generated to a peptide from the first nucleoplasmic region of TMEM120A was confirmed to specifically recognize the protein by its reacting with exogenously expressed TMEM120A fused to GFP in HT1080 fibroblast cells that do not detectably express TMEM120A endogenously (Fig 2A, left panel). This antibody was specific for TMEM120A and failed to recognize TMEM120B similarly overexpressed as a fusion to GFP, though the expression of the fusion protein could clearly be confirmed with anti-GFP antibodies. Attempts to generate antibodies specific to TMEM120B or that would recognize both TMEM120A and TMEM120B were unsuccessful; therefore most of the subsequent experiments were performed using quantitative real-time PCR (qRT-PCR). Both antibody specificity and the ability to achieve a protein knockdown were also confirmed using the TMEM120A antibody with TMEM120A shRNA, where a roughly 90% protein knockdown was achieved (Fig 2A, right panels).

Many NETs have been found to be highly tissue-specific [25,26,28] so the presence of TMEM120A protein in various mouse tissues was investigated using these antibodies (Fig 2B). Only a very weak signal was observed for most tissues whereas a robust band of the expected molecular weight for the endogenous protein was observed in the protein lysate generated from WAT. Two higher molecular weight bands were observed robustly in brain and weakly in heart; however, the sizes of these bands were greater than predicted from the existing annotated exons of the gene sequence. These bands might reflect non-specific binding of the antibody to distinct proteins in these tissues, despite that the peptide sequence used to generate the antibody was unique and the antibody was affinity purified. Alternatively, it could indicate poor gene annotation such that there are additional as yet to be identified exons in the gene. The expected size TMEM120A band was almost undetectable in mouse liver, surprisingly, as it was originally identified in mouse liver NEs [27]. This and the weak bands in other tissues might be explained by its presence in fat within the liver and these other tissues, as it was only identified in the mass spectrometry of liver NEs with a single peptide and so was probably of low abundance.

As our attempts to generate TMEM120B antibodies were unsuccessful and no commercial antibody specific for TMEM120B was available, we were not able to similarly test for the endogenous TMEM120B protein levels in tissues. However, we could compare the expression of the genes encoding TMEM120A and TMEM120B by qRT-PCR in tissues. Both *Tmem120A* and *Tmem120B* genes were highest expressed in inguinal and subcutaneous WAT and in brown adipose tissue (BAT) (Fig 2C). *Tmem120A* was also expressed to low levels in liver whereas *Tmem120B* was virtually undetectable, consistent with our identification of peptides only for TMEM120A in the liver NE proteomics. Surprisingly, the qRT-PCR primers did not detect high levels in brain or heart; therefore the higher molecular weight protein recognized by the TMEM120A antibodies must either lack the exons targeted or be an unrelated protein.

### TMEM120A and B are induced during adipocyte differentiation

**Fig 3:**
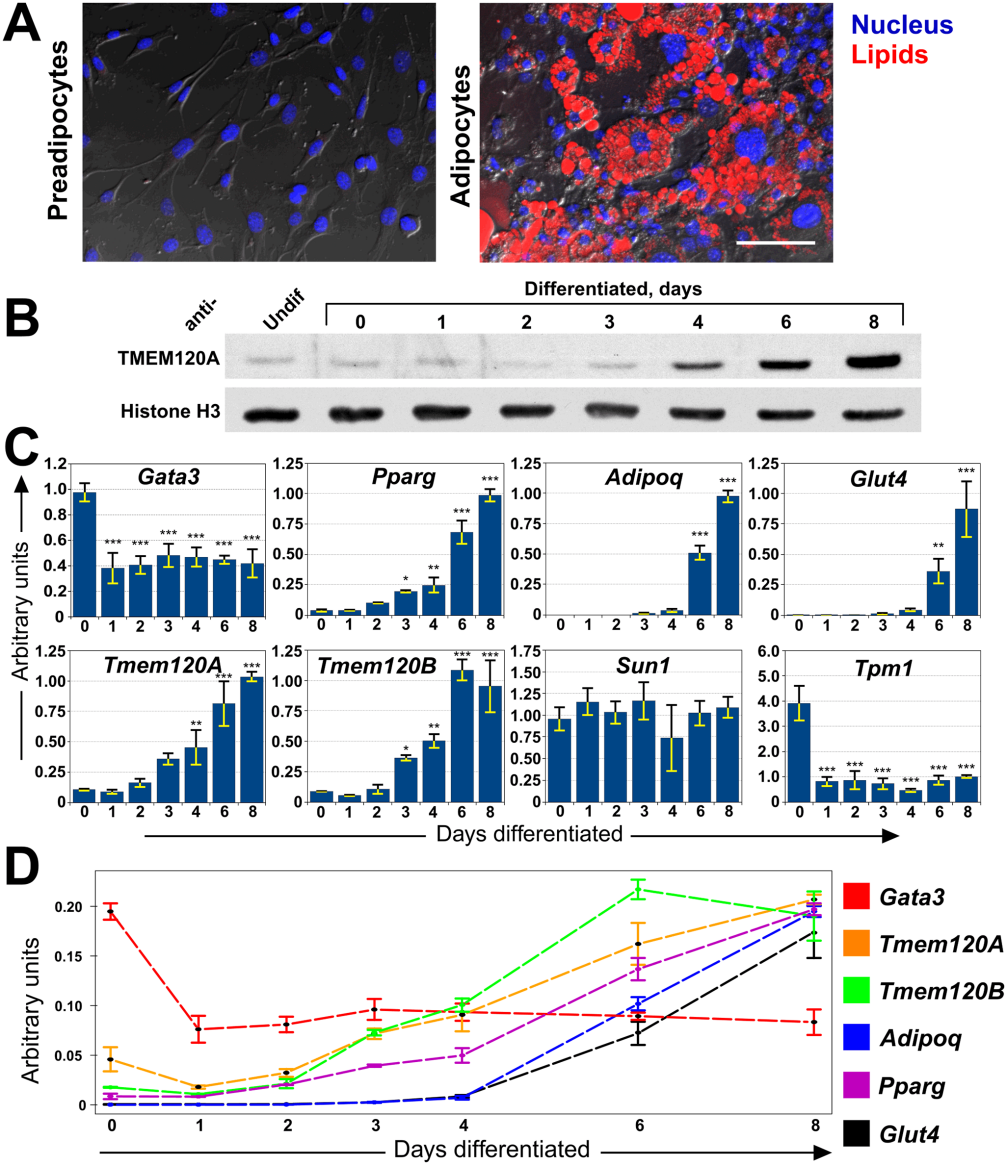
Induction of TMEM120A, TMEM120B and adipogenic markers during adipogenesis. (A) The 3T3-L1 *in vitro* differentiation system. Left panel, 3T3-L1 pre-adipocytes have a normal fibroblast morphology and do not stain with LipidTOX which stains lipid droplets. Right panel, after induction by treatment with iso-butyl methyl xanthine, dexamethasone and insulin the cells begin to accumulate lipid droplets by day 4 and this increases so that by day 8 (shown) the vast majority of cells have generated many large lipid droplets that stain with LipidTOX. Scale bar, 100 μm. (B) Lysates were generated from cells over a timecourse of 3T3-L1 induction and reacted with Tmem120A antibodies or with histone H3 antibodies as a loading control. The protein is induced over time, with the major accumulation between days 3 and 8. (C) RNA was extracted from a timecourse of 3T3-L1 adipogenesis and the relative mRNA levels for *Tmem120A*, *Tmem120B*, *Gata3*, *Pparg*, *AdipoQ*, *Glut4* and controls *Sun1* and *Tpm1* measured by qRT-PCR. Asterisks indicate statistical significance (* p<0.05, ** p<0.01, *** p<0.001). (D) A subset of the same data overlaid as line plots for direct comparison clearly shows the earlier activated *Gata3* dropping prior to activation of the *Tmem120A* and *B* genes which become activated in relative levels at a faster rate than the master regulator *Pparg*.

To further investigate the relationship of TMEM120A with fat, the well-established 3T3-L1 *in vitro* adipogenesis model system was used. 3T3-L1 cells were originally clonally isolated from the NIH 3T3 murine cell line for their ability to accumulate triglycerides [30]. The fibroblast-like 3T3-L1 cells have characteristics of pre-adipocytes and can be induced *in vitro* to form adipocytes characteristic to WAT by a combination of growth arrest and addition of an adipogenic cocktail consisting of insulin, iso-butyl methyl xanthine and dexamethasone [30–32]. With this treatment the cells change their shape and accumulate large cytoplasmic lipid droplets (Fig 3A). Total proteins were extracted from sub-confluent pre-adipocytes or from cells induced to differentiate at various time points. Equal amounts of total protein were resolved on SDS PAGE and TMEM120A protein levels were assessed using histone H3 as a loading control (Fig 3B). While histone H3 levels remained similar throughout differentiation, TMEM120A protein levels increased significantly during differentiation.

As we did not have antibodies specific for TMEM120B, mRNA levels were measured and compared for both *Tmem120A* and *Tmem120B*. RNA was extracted from the same timecourse of 3T3-L1 adipogenesis to be able to relate them to the TMEM120A protein results and the relative mRNA levels were measured for these genes and several adipogenic markers and controls (Fig 3C). As one control, the widely expressed NET SUN1 remained unchanged during 3T3-L1 adipogenic differentiation (p = 0.24). As another control the muscle-specific *Tpm1* (tropomyosin) should be downregulated in adipogenesis because muscle and fat are divergent developmental pathways from the same precursors. Thus, *Tpm1* was also tested and indeed was strongly downregulated during 3T3-L1 adipogenic differentiation (p = 4.22e-08). All ANOVA statistics for this and other figures are available in S1 Table. *Tmem120B* was upregulated during adipogenic differentiation of the 3T3-L1 cells with very similar kinetics to *Tmem120A* (Tmem120a is just missing statistical significance on day 3 of differentiation with p = 0.052). Interestingly, both matched quite closely the induction profile of the central adipogenic transcriptional regulator *Pparg* [39] and were induced much earlier than the frequently used markers of adipogenesis *Adipoq* and *Glut4* (Fig 3C). The *Adipoq* gene product regulates glucose levels and fatty acid oxidation [40,41] while the *Glut4* gene product is a glucose transporter [42].

PPARγ transcriptional cascades are induced by the drug Rosiglitazone with a concomitant upregulation of TMEM120A [43], suggesting that PPARγ could drive TMEM120A expression. However, the matched induction profile between *Pparg*, the gene encoding PPARγ, and *Tmem120A* and *B* in 3T3-L1 adipogenesis would argue that the TMEM120 genes can be induced by an earlier factor. To determine if the resolution of the qRT-PCR was sufficient to distinguish earlier factors, the upstream transcriptional regulator GATA-3 [44] was also assayed for. Indeed *Gata3* was observed to drop early in the 3T3-L1 differentiation just prior to activation of the *Tmem120* genes and *Pparg* (Fig 3C). Replotting the data overlaid as a line plot for direct comparison clearly shows the earlier activation of *Gata3* and further indicates both that *Tmem120A* experienced a similar drop to *Gata3* just before its increase and that the subsequently induced *Tmem120A* and *B* genes increased in relative transcript levels at an even faster rate than *Pparg* (Fig 3D).

### TMEM120A and B are necessary for efficient adipogenesis

**Fig 4:**
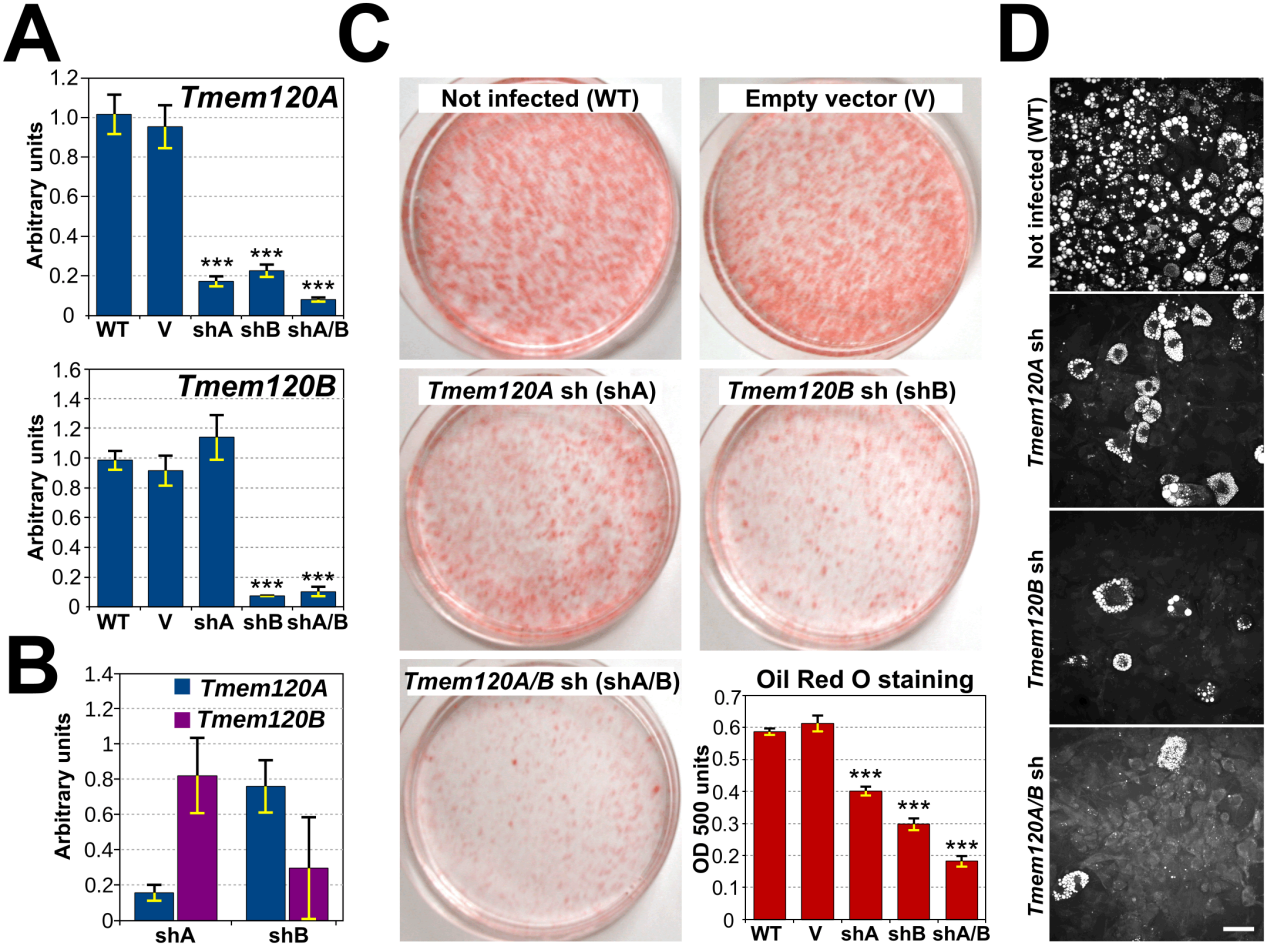
Both TMEM120 proteins are critical for adipogenesis. (A) TMEM120A and TMEM120B were knocked down in 3T3-L1 cells by viral transduction of shRNA constructs both individually and together and the cells were induced pharmacologically to differentiate. RNA was extracted on day 8 and the relative levels of transcripts from both genes using Sun1 as a control was determined by qRT-PCR. Notably TMEM120B knockdown affected both TMEM120B and TMEM120A levels in the cells induced for differentiation. (B) TMEM120B knockdown does not affect the minimal TMEM120A levels in non-differentiated cells. The values plotted represent the ratios of *Tmem120* levels in knockdown cell lines to corresponding levels in cells transduced with empty vector. (C) To follow adipocyte differentiation the cells were stained with Oil Red O, a dye that intercalates in lipid droplets (images from day 12 of differentiation). To quantify the extent of differentiation by this measure, the Oil Red O dye was extracted from the cells with isopropanol and measured in a spectrophotometer (lower right panel, timepoint taken at 8 days differentiation). Both TMEM120 proteins knockdowns reduced lipid droplet accumulation by this measure significantly on their own and both together reduced lipid droplets by roughly 70%. (D) Lipid droplet accumulation was also assessed by CARS microscopy, a method based on Raman spectroscopy that directly highlights these structures without the use of dyes (images taken at 8 days differentiation). Scale bar, 50 μm.

In order to further investigate involvement of the TMEM120 proteins in adipogenesis, 3T3-L1 cell lines stably expressing shRNA constructs to knock down TMEM120A, TMEM120B, or both together were generated. Because 3T3-L1 cells have been reported to exhibit a reduction in their differentiation capacity at passages greater than 15, the lines were generated by viral transduction in freshly thawed early passage cells. These cell lines, as well as control cells expressing the empty shRNA vector and wild-type cells, were grown in parallel to confluency and induced pharmacologically to differentiate using standard conditions. At day 8 of differentiation total RNA was isolated and the relative levels of TMEM120A and TMEM120B mRNA were quantified (normalized to control SUN1) to measure the success of the knockdowns (Fig 4A). The TMEM120A knocked down to less than 30% of the normal levels in the adipocytes (p ≤ 5e-07 for both the untransfected and for the empty vector). Interestingly, the shRNA targeting TMEM120B also reduced TMEM120A mRNA levels (p ≤ 9e-07 for both the untransfected and for the empty vector) in the TMEM120B knockdown despite that the targeting oligo was predicted *in silico* to be unique for TMEM120B (see Materials and Methods). It is unlikely that the shRNA targeting TMEM120B directly targets TMEM120A because in the undifferentiated pre-adipocytes that had lower endogenous TMEM120 levels the shRNA targeting is specific. shA effectively knocks down Tmem120A compared to the empty vector treatment (p = 0.0006) while shB does not (p = 0.24). Correspondingly, shB effectively knocks down Tmem120B compared to the empty vector treatment (p = 0.025) while shA does not (p = 0.67) (Fig 4B). This suggests that the effect of TMEM120B knockdown on levels of TMEM120A is indirect and caused by poor differentiation of TMEM120B knockdown cells.

To test for the effect of loss of the TMEM120 proteins on adipogenesis, the cells were stained with Oil Red O, a dye that preferentially intercalates with the neutral triglycerides and lipids of lipid droplets. The TMEM120A knockdown had visibly less staining than the wild-type and empty vector controls throughout differentiation and this difference was still observable after 12 days post-induction of differentiation (Fig 4C). The TMEM120B knockdown had a more prominent effect on differentiation, so that, despite the reduction also in TMEM120A transcript levels, it was clear that TMEM120B also contributes to adipogenesis. The combined knockdown had a greater effect than either alone, further indicating that both contribute, at least partly redundantly, to adipocyte differentiation. To quantify the loss of differentiation, the Oil Red O was extracted with isopropanol from a triplicate set of plates on day 8 of differentiation. The amount of extracted dye measured by spectrometry should generally be proportional to the amount of lipid droplets and thus provides a measure of adipocyte differentiation. The levels in the TMEM120A single knockdown cells were reduced to 68% compared to cells transduced with empty vector control (p = 3e-07), 49% in the TMEM120B knockdown cells (p = 4e-09) (that also had partial knockdown of TMEM120A), and to roughly 1/3 (31%) in the double knockdown cells (p = 2e-10) (Fig 4C, graph). The amount of lipid droplets could also be observed to drop substantially using CARS microscopy for direct imaging of lipid droplets in live cells without the use of dyes (Fig 4D).

**Fig 5:**
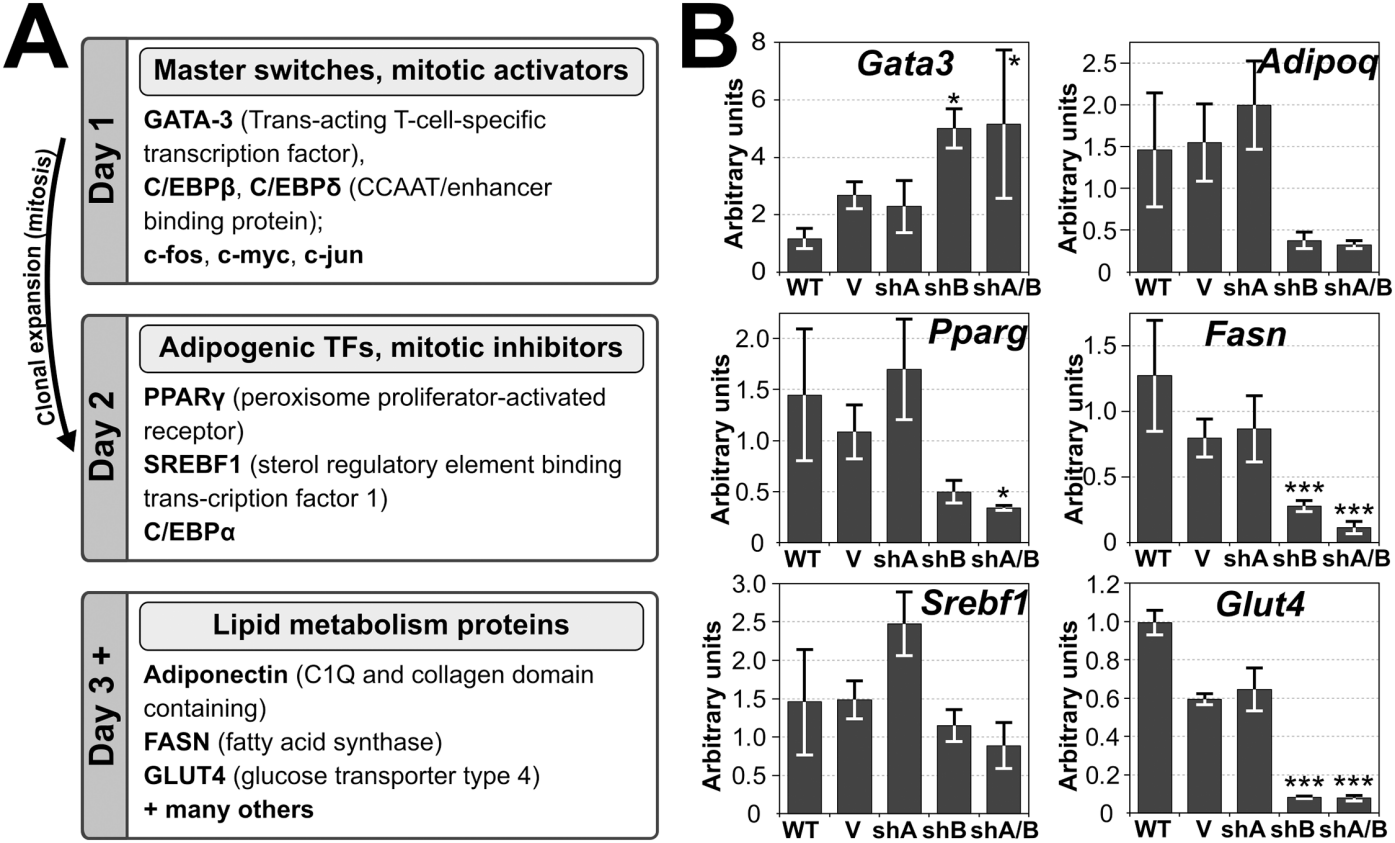
Both early expressed adipogenic transcription factors and late expressed lipid metabolism proteins transcripts are targeted by TMEM120 protein functions. (A) Well-defined protein functional cascades during pharmacologically induced 3T3-L1 differentiation. Early upregulated or downregulated genes include *Gata3*, *Srebf1* and master regulator of adipogenesis, *Pparg*. Later in differentiation, levels of their targets, proteins involved in lipid methabolism, raise. [44,45] (B) RNA extracted from the TMEM120A and TMEM120B knockdown cells at day 8 of the adipogenic differentiation in Fig 4 were analyzed by qRT-PCR for effects on genes known to be important for adipogenesis (e.g. *Gata3*, *Pparg*, *Srebf1*) or fat metabolism (e.g. *Fasn*, *AdipoQ*, *Glut4*). Both NETs had effects, but the inhibitory effect of TMEM120B knockdown was stronger for most of the genes tested.

The reduction in number of lipid droplets within the population could reflect a primary effect on levels of key lipid metabolism proteins or, more generally, adipogenic transcription activators/drivers of differentiation that control the levels of the former (Fig 5A) [44,45]. If the first were the case, only levels of late expressing lipid metabolism proteins would be affected in the TMEM120 knockdown cell lines while levels of early expressing transcription factors would be similar to wild-type cells. If the second were true, both early transcription factors and their targets, late expressing lipid metabolism proteins would differ in TMEM120 knockdown cell lines. To determine this, the RNA isolated from the experiment in Fig 4A at day 8 of differentiation was tested for the levels of transcripts of proteins associated with adipogenic transcription cascades and adipocyte metabolic pathways (Fig 5B). The master switch Gata3 is normally downregulated by day 1 of differentiation and stays down by day 8 (Fig 3C). By contrast, in the Tmem120A/B double knockdown cell line, Gata3 downregulation fails and is expressed much higher than in the wild-type cells at day 8 (Fig 5B p = 0.02). Pparg and Srebf1 are transcription factors normally induced during differentiation, after Gata3 has been downregulated, and stay expressed strongly by day 8 (Figs 3C and 5A). In the Tmem120A/B double knockdown cell line, however, Pparg but not Srebf1 fails to be upregulated (Fig 5B p = 0.04 and 0.47 respectively). Later enzymatic factors Glut4 and Fasn also fail to be upregulated in the Tmem120A/B double knockdown cell line (Fig 5B, p = 3.2e-08 and 7.5e-04 respectively) and Adipoq narrowly missed statistical significance (p = 0.06). Thus, knockdown of both Tmem120 genes stalls adipogenesis likely by affecting some early transcription factors. In some cases Tmem120A and/or Tmem120B knockdown alone had statistically significant effects (S1 Table).

**Fig 6:**
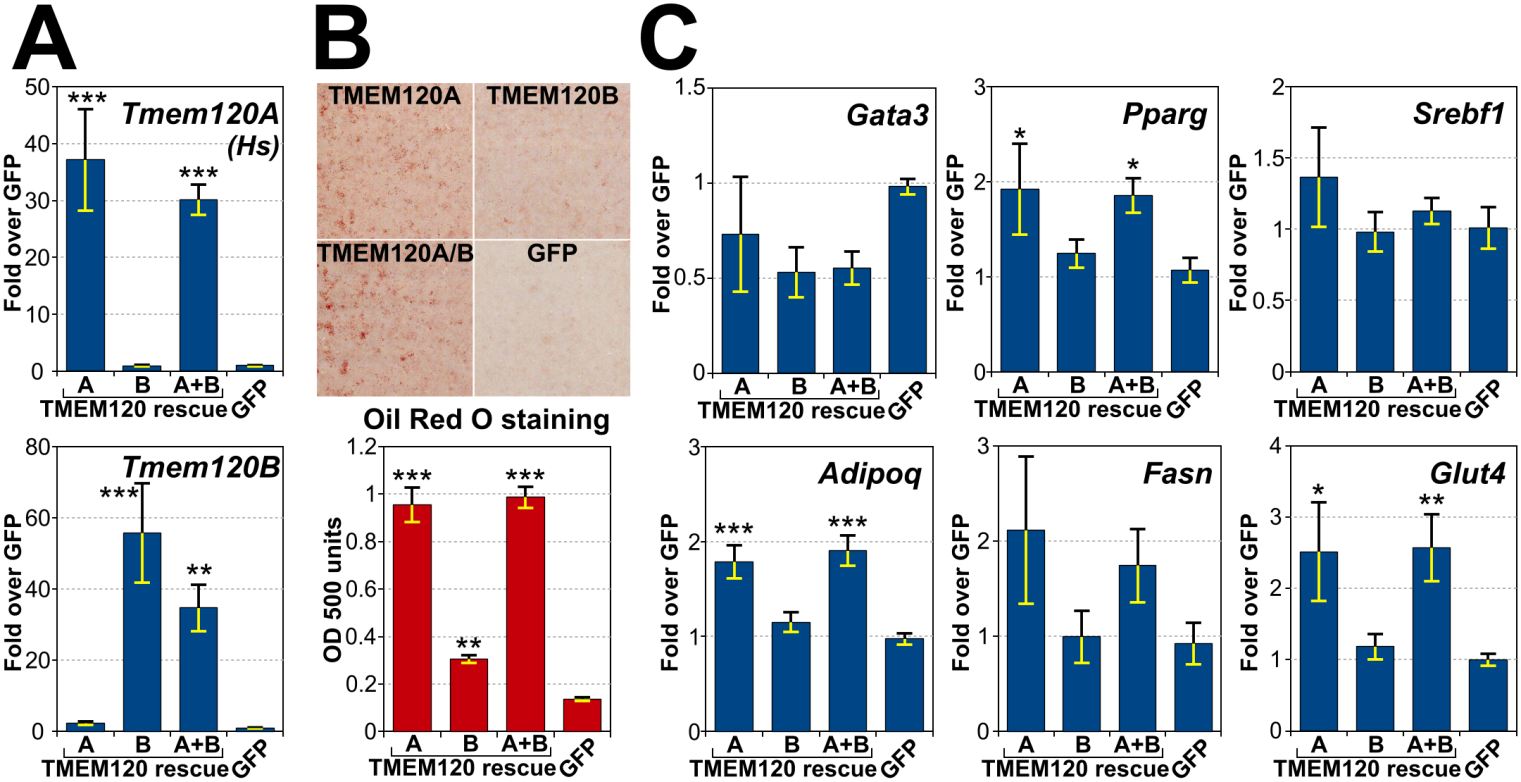
Reintroduction of TMEM120 proteins rescues adipogenesis. Either shRNA resistant TMEM120A, TMEM120B, the combination of the two, or a GFP control were expressed in *Tmem120A/B* double knockdown cells and the cells were induced pharmacologically to differentiate. (A) Levels of human *Tmem120A* and mouse *Tmem120B* transcripts were assessed in the rescue cell lines from 8 days post adipogenic induction. (B) Oil Red O staining of the rescue cell lines. Top panel, cells stained with Oil Red O. Bottom panel, quantification by spectrophotometry at OD 500 of the dye extracted from cells. (C) RNA extracted from cells in this experiment was used to measure transcript levels of important adipogenic transcription factors and lipid metabolism proteins.

In general, Tmem120A knockdown appeared to have less of an effect on adipogenesis than Tmem120B knockdown; however, the indirect effects of *Tmem120B* knockdown on levels of *Tmem120A* in differentiating cells may have masked the importance of TMEM120A in adipogenesis. To address this, the TMEM120 proteins were compared for their ability to rescue adipogenic and gene expression defects in the double knockdown cells. Tmem120A/B double knockdown cells were transfected with shRNA resistant human *Tmem120A*, shRNA resistant mouse *Tmem120B*, or a combination of the two. As a control, the same cell line expressing GFP was used. Levels of TMEM120A and B were measured in the resulting cell lines, confirming specific overexpression of corresponding transcripts (Fig 6A). The double knockdown cells expressing the various proteins were induced to differentiate pharmacologically using the standard protocol, and adipogenesis was measured by Oil Red O staining (Fig 6B). All rescues were statistically significant compared to the GFP alone (all three p < 0.006), but, surprisingly, TMEM120A alone rescued to ~95% of the level of both TMEM120A and B while TMEM120B alone only rescued to ~30%. Testing individual defects in transcript levels in the four early transcription factors and lipid metabolism proteins that had significant effects in the double knockdown cells the TMEM120A rescued in three of them and the fourth was close with p = 0.055 while TMEM120B did not have statistically significant effects (Fig 6C).

### TMEM120A can form homo-oligomers and hetero-dimerize with TMEM120B

**Fig 7:**
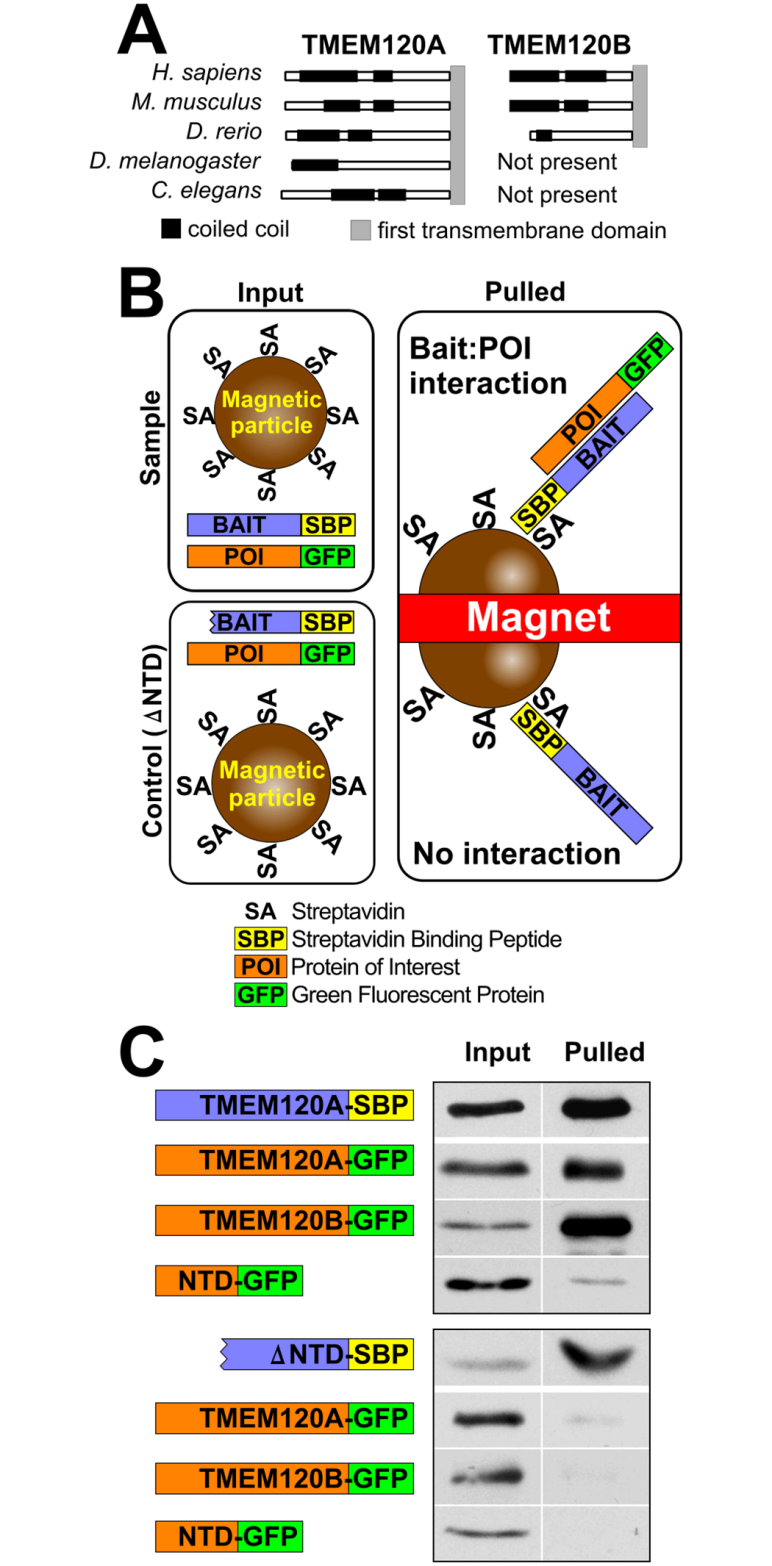
Oligomerization of TMEM120A and B. (A) Coiled-coil domains are strongly predicted within the N-terminal regions of both TMEM120A and B using the COILS2 server for all organisms having both TMEM120 genes and predicted in the single protein for organisms having only one TMEM120 gene. (B) Schematic of assay and expected outcomes. Cells co-express streptavidin binding protein (SBP) fused to one TMEM120 protein and GFP fused to the same or another TMEM120 protein. The cells are lysed and the SBP fusion proteins are pulled from the lysate with streptavidin conjugated to magnetic beads. If the other TMEM120 protein fused to GFP is pulled out together with the SBP tagged protein it indicates interaction between the two TMEM120 proteins expressed. As a negative sontrol, TMEM120A-SBP fusion lacking predicted coil-coiled domains (deleted 2–105 amino acids, ΔNTD) was used. (C) Proteins pulled out of the lysates by the magnetic streptavidin coated beads were analyzed by Western blot using antibodies to GFP to detect the GFP fusions and streptavidin to test the SBP fusions. This confirmed both hetero- and homo-dimer formation are possible among TMEM120 proteins. The soluble N-terminal fragment containing the predicted coiled coils also interacted with the full-length protein, but not the ΔNTD mutant.

The indication that TMEM120A and B have overlapping functions in adipogenesis raised the possibility that the proteins could function together in a complex. Both TMEM120A and TMEM120B have strongly predicted coil-coiled domains within their N-termini (Fig 7A). As this domain often mediates homo-oligomerization, we decided to first test the possibility that TMEM120A can interact with itself to form oligomers. 3T3-L1 cells were co-transfected with plasmids encoding TMEM120A-SBP and TMEM120A-GFP fusion proteins. As a negative control, the same fusion with deleted 2–105 amino acids (lacking all predicted coil-coiled domains), ΔNTD, was used. SBP fusion proteins were then pulled down using streptavidin coated magnetic particles (Fig 7B). Pull down was confirmed by probing pulled material with anti-TMEM120A antibody in case of full-length protein, and anti-SBP antibody in case of N-terminally deleted protein (Fig 7C). We then probed pulled material with anti-GFP antibody, and it clearly showed enrichment of TMEM120A-GFP fusion protein, while it was not found in the pulled material of a control sample. Next we tested if TMEM120A is able to oligomerize with TMEM120B. In a similar experiment, we co-expressed TMEM120A-SBP or its ΔNTD mutant, with TMEM120B-GFP fusion proteins, pulled down SBP proteins and found that TMEM120B-GFP is also enriched in the pulled material of full length SBP fusion, but not in the negative control (Fig 7C). Additionally, a soluble N-terminal fragment of TMEM120A was tested that includes the predicted coiled-coils. This was also able to oligomerize with full-length TMEM120A but not its Δ,NTD mutant although the amount recovered was lower than for the full-length proteins (Fig 7C). This could be easily explained by the proteins being in different phases inside the cell—soluble N-terminal fragment occupying cytoplasm and nucleoplasm, while SBP fusion proteins are membrane-bound. Alternatively, this could indicate that other regions of the protein are involved in the interaction. In either case the coiled coils are most likely to mediate the oligomerization. These results show that TMEM120A can form both homo-oligomers and also can form hetero-oligomers with TMEM120B.

## Discussion

Here we have identified a function for the TMEM120A and B proteins, products of the *Tmem120A* and *B* genes, in adipogenesis. Although this is the first direct testing of this function, it is not surprising that these proteins contribute to adipogenesis and/or fat metabolism because *Tmem120A* has now been linked in a variety of transcriptome studies to these functions by its up- or down-regulation in differing conditions. One transcriptome study of visceral fat in obese Pima Indians found a roughly 2-fold reduction in *Tmem120A* transcript levels compared to the non-obese population (*GDS1495/48102_s_at*). Less directly, levels of *Tmem120A* transcripts were altered in a co-expression network analysis of fatty acid metabolism in liver [46], in response to altering the liver fatty acid transcription coactivator mediator subunit MED1 [47], and in comparing hormone therapy regimens in postmenopausal women [48]. Moreover, *Tmem120A* was found to be upregulated in 3T3-L1 cells treated with the anti-diabetic drug rosiglitazone [43], further indicating a function in aspects of fat metabolism. Here we have not only identified specific defects in lipid droplet accumulation in adipocyte differentiation upon TMEM120A knockdown, but have found that it functions both together and somewhat redundantly with TMEM120B. This now confirms a role for TMEM120 proteins in adipogenesis.

While all the publications mentioning the *Tmem120A* gene listed above have been transcriptome studies linking it to aspects of fat metabolism, it has also come up in transcriptome studies seemingly unrelated to fat metabolism. The original NCBI annotation for TMEM120A labeled it TMPIT for transmembrane protein induced by TNFα because it appeared in a transcriptome list of genes upregulated by TNFα in endothelial cells, however, the details of this study were not published. *Tmem120A* also appeared in a list of genes differentially expressed in HTLV-1 infected CD4^+^ T cells [49] and a list of genes changing at the E10.5 stage of limb development [50]. While the former is harder to explain in the context of fat, it is possible that the latter reflects the beginnings of fat differentiation. There was also one report linking *Tmem120A* to fat metabolism, but not in fat itself. In chickens, hypothalamus transcriptome profiling—searching for systemic metabolic contributors to obesity—found *Tmem120A* down in lean chickens compared to fat chickens [51]. This is particularly interesting in our observation of a higher molecular weight band than could be accounted for by the splice variants currently in public databases appearing in the brain protein lysates.

Though we have clearly confirmed both TMEM120A and TMEM120B to be NE proteins, TMEM120A was recently listed among a large number of proteins identified in a proteomic identification of plasma membrane components of 3T3-L1 adipocytes [52]. Although we have often observed some fraction of NETs when overexpressed in the ER [29] and indeed half of the splice variants predicted at NCBI for TMEM120B have predicted ER membrane retention predictions, we did not specifically observe TMEM120A in the plasma membrane. This might just reflect contamination considering how strongly TMEM120A is induced during adipogenesis or it might reflect a separate unique function for a small subpopulation at the plasma membrane as it has been proposed that roughly 40% of cellular proteins have multiple subcellular localizations and functions [53]. Regardless, the strong effects on fat differentiation and/or metabolism likely reflect the action of the dominant nuclear pool.

In this light it seems unlikely that the protein would contribute to fat metabolism from an enzymatic function in the cytoplasm. More likely the action of TMEM120A and B is related to regulation of gene expression through a recently reported function for TMEM120A in gene positioning [54]. Such a function is consistent with the range of both differentiation and metabolic pathway genes affected by its knockdown. This type of a function would also be more consistent with its lack of homologs in lower organisms such as fungi as if it were involved in metabolism itself it might be expected to be more widely conserved. The idea of a function in regulation of gene expression is also consistent with the observation that a TMEM120 homolog in *Oryza sativa indica* (Rice) is fused to a Myb-like DNA-binding domain.

Most NETs bind lamins and it was reported earlier this year that TMEM120B is one of hundreds of proteins found to interact with lamin A in a 2-hybrid screen [55]. The inner nuclear membrane localization that we previously determined for TMEM120A and its resistance in the NE to a pre-extraction with detergent are consistent with TMEM120A also binding to lamins, though the NE distribution of TMEM120A was not lost in lamin A knockout fibroblasts [29]. Thus it may be that TMEM120 proteins can bind to more than one lamin subtype. In either case the combination of tissue-specificity of the TMEM120 proteins and the indication of their binding to lamin A that is mutated in familial partial lipodystrophy Dunnigan-type and several other syndromes with associated lipodystrophy [16–20] suggests the possibility that the TMEM120 proteins contribute to mediating the fat-specific pathology in these disorders.

## Supporting Information

S1 Table Statistical analysis of qPCR and OilRedO data. Analysis was performed using ANOVA followed by post-hoc Tukey HSD correction.
(XLSX)

## References

[1] WHO. Obesity and overweight [Internet]. 2011. Available: http://www.who.int.mediacentre/factsheets/fs311/en/

[2] Fall T, Ingelsson E. Genome-wide association studies of obesity and metabolic syndrome. Mol Cell Endocrinol. 2014;382: 740–757. 10.1016/j.mce.2012.08.018 22963884

[3] Willyard C. Heritability: The family roots of obesity. Nature. 2014;508: S58–60. 10.1038/508S58a 24740129

[4] Ali AT, Hochfeld WE, Myburgh R, Pepper MS. Adipocyte and adipogenesis. Eur J Cell Biol. Elsevier GmbH.; 2013;92: 229–236. 10.1016/j.ejcb.2013.06.001 23876739

[5] De Las Heras JI, Meinke P, Batrakou DG, Srsen V, Zuleger N, Kerr AR, et al Tissue specificity in the nuclear envelope supports its functional complexity. Nucleus. 2013/11/12 ed. 2013;4: 460–477. doi:26872 [pii] 10.4161/nucl.26872 24213376 PMC3925691

[6] Maraldi NM, Capanni C, Cenni V, Fini M, Lattanzi G. Laminopathies and lamin-associated signaling pathways. J Cell Biochem. 2011/03/15 ed. 2011;112: 979–992. 10.1002/jcb.22992 21400569

[7] Muchir A, Pavlidis P, Bonne G, Hayashi YK, Worman HJ. Activation of MAPK in hearts of EMD null mice: similarities between mouse models of X-linked and autosomal dominant Emery Dreifuss muscular dystrophy. Hum Mol Genet. 2007/06/15 ed. 2007;16: 1884–1895. 10.1093/hmg/ddm137 17567779

[8] Muchir A, Pavlidis P, Decostre V, Herron AJ, Arimura T, Bonne G, et al Activation of MAPK pathways links LMNA mutations to cardiomyopathy in Emery-Dreifuss muscular dystrophy. J Clin Invest. 2007/04/21 ed. 2007;117: 1282–1293. 10.1172/JCI29042 17446932 PMC1849984

[9] Clouthier DE, Comerford SA, Hammer RE. Hepatic fibrosis, glomerulosclerosis, and a lipodystrophy-like syndrome in PEPCK-TGF-beta1 transgenic mice. J Clin Invest. 1998/02/12 ed. 1997;100: 2697–2713. 10.1172/JCI119815 9389733 PMC508473

[10] Ross SE, Hemati N, Longo KA, Bennett CN, Lucas PC, Erickson RL, et al Inhibition of adipogenesis by Wnt signaling. Science (80-). 2000;289: 950–953. 10.1126/science.289.5481.950 10937998

[11] Osada S, Ohmori SY, Taira M. XMAN1, an inner nuclear membrane protein, antagonizes BMP signaling by interacting with Smad1 in Xenopus embryos. Development. 2003;130: 1783–1794. Available: http://www.ncbi.nlm.nih.gov/entrez/query.fcgi?cmd=Retrieve&db=PubMed&dopt=Citation&list_uids=12642484 12642484 10.1242/dev.00401

[12] Pan D, Estévez-Salmerón LD, Stroschein SL, Zhu X, He J, Zhou S, et al The integral inner nuclear membrane protein MAN1 physically interacts with the R-Smad proteins to repress signaling by the transforming growth factor-{beta} superfamily of cytokines. J Biol Chem. 2005;280: 15992–16001. 10.1074/jbc.M411234200 15647271

[13] Raju GP, Dimova N, Klein PS, Huang HC. SANE, a novel LEM domain protein, regulates bone morphogenetic protein signaling through interaction with Smad1. J Biol Chem. 2003;278: 428–437. Available: http://www.ncbi.nlm.nih.gov/entrez/query.fcgi?cmd=Retrieve&db=PubMed&dopt=Citation&list_uids=12393873 12393873 10.1074/jbc.M210505200

[14] Bourgeois B, Gilquin B, Tellier-Lebegue C, Ostlund C, Wu W, Perez J, et al Inhibition of TGF-beta signaling at the nuclear envelope: characterization of interactions between MAN1, Smad2 and Smad3, and PPM1A. Sci Signal. 2013/06/20 ed. 2013;6: ra49. doi:6/280/ra49 [pii] 10.1126/scisignal.2003411 23779087 PMC3843637

[15] Tilgner K, Wojciechowicz K, Jahoda C, Hutchison C, Markiewicz E. Dynamic complexes of A-type lamins and emerin influence adipogenic capacity of the cell via nucleocytoplasmic distribution of beta-catenin. J Cell Sci. 2009/01/08 ed. 2009;122: 401–413. doi:jcs.026179 [pii] 10.1242/jcs.026179 19126678 PMC2724731

[16] Cao H, Hegele RA. Nuclear lamin A/C R482Q mutation in Canadian kindreds with Dunnigan- type familial partial lipodystrophy. Hum Mol Genet. 2000;9: 109–112. Available: http://www.ncbi.nlm.nih.gov/cgi-bin/Entrez/referer?http://www.ncbi.nlm.nih.gov/htbin-post/Omim/getmim?field=medline_uid&search=10587585 10587585 10.1093/hmg/9.1.109

[17] Shackleton S, Lloyd DJ, Jackson SNJ, Evans R, Niermeijer MF, Singh BM, et al LMNA, encoding lamin A/C, is mutated in partial lipodystrophy. Nat Genet. 2000;24: 153–156. 10655060 10.1038/72807

[18] Novelli G, Muchir A, Sangiuolo F, Helbling-Leclerc A, D’Apice MR, Massart C, et al Mandibuloacral dysplasia is caused by a mutation in LMNA-encoding lamin A/C. Am J Hum Genet. 2002;71: 426–431. 12075506 10.1086/341908 PMC379176

[19] Csoka AB, Cao H, Sammak PJ, Constantinescu D, Schatten GP, Hegele RA. Novel lamin A/C gene (LMNA) mutations in atypical progeroid syndromes. J Med Genet. 2004;41: 304–308. Available: http://www.ncbi.nlm.nih.gov/entrez/query.fcgi?cmd=Retrieve&db=PubMed&dopt=Citation&list_uids=15060110 15060110 10.1136/jmg.2003.015651 PMC1735741

[20] Chen L, Lee L, Kudlow BA, Dos Santos HG, Sletvold O, Shafeghati Y, et al LMNA mutations in atypical Werner’s syndrome. Lancet. 2003;362: 440–445. Available: http://www.ncbi.nlm.nih.gov/entrez/query.fcgi?cmd=Retrieve&db=PubMed&dopt=Citation&list_uids=12927431 12927431 10.1016/S0140-6736(03)14069-X

[21] Lloyd DJ, Trembath RC, Shackleton S, Le L. A novel interaction between lamin A and SREBP1: implications for partial lipodystrophy and other laminopathies. Hum Mol Genet. 2002;11: 769–777. Available: http://www.ncbi.nlm.nih.gov/entrez/query.fcgi?cmd=Retrieve&db=PubMed&dopt=Citation&list_uids=11929849 11929849 10.1093/hmg/11.7.769

[22] Kim JB, Spiegelman BM. ADD1/SREBP1 promotes adipocyte differentiation and gene expression linked to fatty acid metabolism. Genes Dev. 1996;10: 1096–1107. 10.1101/gad.10.9.1096 8654925

[23] Kim JB, Wright HM, Wright M, Spiegelman BM. ADD1/SREBP1 activates PPARgamma through the production of endogenous ligand. Proc Natl Acad Sci U S A. 1998;95: 4333–4337. Available: http://www.ncbi.nlm.nih.gov/pubmed/9539737 9539737 10.1073/pnas.95.8.4333 PMC22489

[24] Boguslavsky RL, Stewart CL, Worman HJ. Nuclear lamin A inhibits adipocyte differentiation: Implications for Dunnigan-type familial partial lipodystrophy. Hum Mol Genet. 2006;15: 653–663. 10.1093/hmg/ddi480 16415042

[25] Korfali N, Wilkie GS, Swanson SK, Srsen V, Batrakou DG, Fairley E a L, et al The leukocyte nuclear envelope proteome varies with cell activation and contains novel transmembrane proteins that affect genome architecture. Mol Cell Proteomics. 2010/08/10 ed. 2010;9: 2571–2585. doi:M110.002915 [pii] 10.1074/mcp.M110.002915 20693407 PMC3101955

[26] Korfali N, Wilkie GS, Swanson SK, Srsen V, de Las Heras J, Batrakou DG, et al The nuclear envelope proteome differs notably between tissues. Nucleus. 2012/09/20 ed. 2012;3: 552–564. doi:22257 [pii] 10.4161/nucl.22257 22990521 PMC3515538

[27] Schirmer EC, Florens L, Guan T, Yates JR 3rd, Gerace L. Nuclear membrane proteins with potential disease links found by subtractive proteomics. Science (80-). 2003;301: 1380–1382. 10.1126/science.1088176 12958361

[28] Wilkie GS, Korfali N, Swanson SK, Malik P, Srsen V, Batrakou DG, et al Several novel nuclear envelope transmembrane proteins identified in skeletal muscle have cytoskeletal associations. Mol Cell Proteomics. 2010/09/30 ed. 2011;10: M110 003129. doi:M110.003129 [pii] 10.1074/mcp.M110.003129 PMC3016689 20876400

[29] Malik P, Korfali N, Srsen V, Lazou V, Batrakou DG, Zuleger N, et al Cell-specific and lamin-dependent targeting of novel transmembrane proteins in the nuclear envelope. Cell Mol Life Sci. 2010/01/22 ed. 2010;67: 1353–1369. 10.1007/s00018-010-0257-2 20091084 PMC2839517

[30] Green H, Kehinde O. Sublines of mouse 3T3 cells that accumulate lipid. Cell. 1974;1: 113–116.

[31] Green H, Kehinde O. An established preadipose cell line and its differentiation in culture. II. Factors affecting the adipose conversion. Cell. 1975/05/01 ed. 1975;5: 19–27. doi:0092-8674(75)90087-2 [pii] 165899 10.1016/0092-8674(75)90087-2

[32] Russell TR, Ho R. Conversion of 3T3 fibroblasts into adipose cells: triggering of differentiation by prostaglandin F2alpha and 1-methyl-3-isobutyl xanthine. Proc Natl Acad Sci U S A. 1976;73: 4516–4520. Available: http://www.ncbi.nlm.nih.gov/pubmed/188043 188043 10.1073/pnas.73.12.4516 PMC431523

[33] Follenzi A, Ailles LE, Bakovic S, Geuna M, Naldini L. Gene transfer by lentiviral vectors is limited by nuclear translocation and rescued by HIV-1 pol sequences. 2000;25: 217–222. 10835641 10.1038/76095

[34] Samejima K, Ogawa H, Cooke CA, Hudson DF, Macisaac F, Ribeiro SA, et al A promoter-hijack strategy for conditional shutdown of multiply spliced essential cell cycle genes. Proc Natl Acad Sci U S A. United States; 2008;105: 2457–2462. 10.1073/pnas.0712083105 18263736 PMC2268158

[35] Pfaffl MW. A new mathematical model for relative quantification in real-time RT-PCR. Nucleic Acids Res. England; 2001;29: e45 11328886 10.1093/nar/29.9.e45 PMC55695

[36] R Development Core Team. R: A Language and Environment for Statistical Computing [Internet]. Vienna, Austria; 2008 Available: http://www.r-project.org

[37] Zumbusch A, Holtom GR, Xie XS. Three-Dimensional Vibrational Imaging by Coherent Anti-Stokes Raman Scattering. Phys Rev Lett. American Physical Society; 1999;82: 4142–4145. 10.1103/PhysRevLett.82.4142

[38] Mouras R, Bagnaninchi P, Downes A, Muratore M, Elfick A. Non linear optical microscopy of adipose-derived stem cells induced towards osteoblasts and adipocytes. Proc SPIE. 2011;8086 10.1117/12.889780 PMC3272462 22318871

[39] Tontonoz P, Hu E, Spiegelman BM. Stimulation of Adipogenesis in Fibroblasts by PPARy2, a Lipid-Activated Transcription. 1994;79: 1147–1156. 8001151 10.1016/0092-8674(94)90006-x

[40] Diez JJ, Iglesias P. The role of the novel adipocyte-derived hormone adiponectin in human disease. Eur J Endocrinol. 2003;148: 293–300. Available: http://www.ncbi.nlm.nih.gov/pubmed/12611609 12611609 10.1530/eje.0.1480293

[41] Yamauchi T, Kamon J, Minokoshi Y, Ito Y, Waki H, Uchida S, et al Adiponectin stimulates glucose utilization and fatty-acid oxidation by activating AMP-activated protein kinase. Nat Med. 2002;8: 1288–1295. 10.1038/nm788 12368907

[42] Bryant NJ, Govers R, James DE. Regulated transport of the glucose transporter GLUT4. Nat Rev Mol Cell Biol. 2002;3: 267–277. 10.1038/nrm782 11994746

[43] Haakonsson AK, Stahl Madsen M, Nielsen R, Sandelin A, Mandrup S. Acute genome-wide effects of rosiglitazone on PPARgamma transcriptional networks in adipocytes. Mol Endocrinol. 2013/07/26 ed. 2013;27: 1536–1549. doi:me.2013-1080 [pii] 10.1210/me.2013-1080 23885096 PMC5415231

[44] Tong Q, Dalgin G, Xu H, Ting CN, Leiden JM, Hotamisligil GS. Function of GATA transcription factors in preadipocyte-adipocyte transition. Science (80-). 2000;290: 134–138. Available: http://www.ncbi.nlm.nih.gov/pubmed/11021798 11021798 10.1126/science.290.5489.134

[45] Ntambi JM, Young-Cheul K. Adipocyte differentiation and gene expression. J Nutr. UNITED STATES; 2000;130: 3122S–3126S. 11110885 10.1093/jn/130.12.3122S

[46] Liu W, Ye H. Co-expression network analysis identifies transcriptional modules in the mouse liver. Mol Genet Genomics. 2014; 10.1007/s00438-014-0859-8 24816893

[47] Bai L, Jia Y, Viswakarma N, Huang J, Vluggens A, Wolins NE, et al Transcription coactivator mediator subunit MED1 is required for the development of fatty liver in the mouse. Hepatology. 2011;53: 1164–1174. 10.1002/hep.24155 21480322 PMC3076129

[48] Dahm AE, Eilertsen AL, Goeman J, Olstad OK, Ovstebo R, Kierulf P, et al A microarray study on the effect of four hormone therapy regimens on gene transcription in whole blood from healthy postmenopausal women. Thromb Res. 2012;130: 45–51. 10.1016/j.thromres.2011.12.009 22217510

[49] Pinto MT, Malta TM, Rodrigues ES, Pinheiro DG, Panepucci RA, Malmegrim de Farias KC, et al Genes Related to Antiviral Activity, Cell Migration, and Lysis Are Differentially Expressed in CD4(+) T Cells in Human T Cell Leukemia Virus Type 1-Associated Myelopathy/Tropical Spastic Paraparesis Patients. AIDS Res Hum Retroviruses. 2014;30: 610–622. 10.1089/AID.2013.0109 24041428 PMC4046198

[50] Taher L, Collette NM, Murugesh D, Maxwell E, Ovcharenko I, Loots GG. Global gene expression analysis of murine limb development. PLoS One. 2011;6: e28358 10.1371/journal.pone.0028358 22174793 PMC3235105

[51] Byerly MS, Simon J, Cogburn LA, Le Bihan-Duval E, Duclos MJ, Aggrey SE, et al Transcriptional profiling of hypothalamus during development of adiposity in genetically selected fat and lean chickens. Physiol Genomics. 2010/04/08 ed. 2010;42: 157–167. doi:physiolgenomics.00029.2010 [pii] 10.1152/physiolgenomics.00029.2010 20371548 PMC3032285

[52] Prior MJ, Larance M, Lawrence RT, Soul J, Humphrey S, Burchfield J, et al Quantitative proteomic analysis of the adipocyte plasma membrane. J Proteome Res. 2011;10: 4970–4982. 10.1021/pr200446r 21928809

[53] Foster LJ, de Hoog CL, Zhang Y, Xie X, Mootha VK, Mann M. A mammalian organelle map by protein correlation profiling. Cell. 2006;125: 187–199. Available: http://www.ncbi.nlm.nih.gov/entrez/query.fcgi?cmd=Retrieve&db=PubMed&dopt=Citation&list_uids=16615899 16615899 10.1016/j.cell.2006.03.022

[54] Zuleger N, Boyle S, Kelly DA, de Las Heras JI, Lazou V, Korfali N, et al Specific nuclear envelope transmembrane proteins can promote the location of chromosomes to and from the nuclear periphery. Genome Biol. 2013/02/19 ed. BioMed Central Ltd; 2013;14: R14. doi:gb-2013-14-2-r14 [pii] 10.1186/gb-2013-14-2-r14 23414781 PMC4053941

[55] Dittmer TA, Sahni N, Kubben N, Hill DE, Vidal M, Burgess RC, et al Systematic identification of pathological lamin A interactors. Mol Biol Cell. 2014;25: 1493–1510. 10.1091/mbc.E14-02-0733 24623722 PMC4004598

